# Early onset of effect following galcanezumab treatment in patients with previous preventive medication failures

**DOI:** 10.1186/s10194-021-01230-w

**Published:** 2021-03-25

**Authors:** Todd J. Schwedt, Dulanji K. Kuruppu, Yan Dong, Katherine Standley, Laura Yunes-Medina, Eric Pearlman

**Affiliations:** 1 Department of Neurology, Mayo Clinic, Phoenix, AZ USA; 2 Eli Lilly and Company, Indianapolis, IN USA; 3 Florida Medical Clinic, Wesley Chapel, FL USA

**Keywords:** Galcanezumab, Migraine, Preventive failure, Early onset, Calcitonin gene-related peptide, CGRP

## Abstract

**Background:**

Galcanezumab is a monoclonal antibody (mAb) that binds calcitonin gene-related peptide (CGRP) and is indicated for the preventive treatment of migraine. Galcanezumab demonstrated early onset of effect in patients with migraine but it is unknown whether the same holds true for patients who have not benefited from multiple prior migraine preventives.

**Methods:**

Patients with episodic or chronic migraine from a 3-month, randomized, double-blind, placebo-controlled, phase 3b study (CONQUER) who had 2 to 4 migraine preventive medication category failures in the past 10 years were randomized 1:1 to placebo (*N* = 230) or galcanezumab 120 mg/month (240 mg loading dose; *N* = 232). In this post-hoc analysis, change from baseline in number of monthly and weekly migraine headache days was assessed. Monthly onset of effect was the earliest month at which significant improvement with galcanezumab compared to placebo was achieved and maintained at all subsequent months. Weekly onset was the initial week at which statistical separation was achieved and maintained at all subsequent weeks during that month. Proportion of patients with migraine headache days in the first week of treatment, and patients achieving ≥50%, ≥75%, and 100% response by month and week were also assessed.

**Results:**

Galcanezumab-treated patients had a significantly greater reduction in monthly migraine headache days starting at month 1, which remained significant for all subsequent months compared to placebo (all *p* ≤ 0.0001, month 1 mean change from baseline: placebo − 0.7; galcanezumab − 4.0). Weekly migraine headache days was significantly reduced in galcanezumab-treated patients starting at week 1 and continued for each subsequent week of month 1 compared to placebo (all *p* < 0.01, week 1 mean change from baseline: placebo − 0.2; galcanezumab − 1.1). A significantly smaller percentage of patients had a migraine headache on the first day after galcanezumab treatment compared to placebo (28.4% vs 39.2%) and at each subsequent day during week 1 (all *p* < 0.05). A greater proportion of galcanezumab-treated patients achieved ≥50%, ≥75%, and 100% response at months 1–3 (all *p* < 0.05) and at weeks 1–4 of month 1 compared to placebo (all *p* < 0.01).

**Conclusion:**

Galcanezumab showed early onset of effect beginning the day after treatment initiation in patients who had not previously benefited from migraine preventive treatments.

**Trial registration:**

ClinicalTrials.gov, NCT03559257. Registered 18 June 2018.

## Background

Migraine is a neurological disease characterized by moderate to severe headaches often accompanied by nausea, vomiting, photophobia, and phonophobia [1]. It is estimated that migraine has a global prevalence of 15% [2]. It has been shown to interfere with occupational, household, family, and social responsibilities [3]. In a recent analysis of the Global Burden of Disease Study 2017, migraine was the second leading cause of years lost to disability [2].

Guidelines state that patients who experience four or more monthly migraine headache days and those who have migraine attacks that cause significant interference with their daily routines despite acute treatment should be offered a preventive therapy [4]. While 40% of patients could benefit from preventive therapy, only 13% of patients use preventive medication [5]. Some oral preventive therapies involve a slow titration schedule and take time to show benefit [6]. Many patients discontinue or switch preventive treatment due to inadequate efficacy or safety/tolerability [7]. More than one-half of patients who receive an oral standard-of-care migraine preventive therapy discontinue its use within 6 months [8, 9]. To increase adherence and improve patient outcomes, it is important to have a medication with an early onset of effect and a favorable tolerability profile [9, 10].

Galcanezumab is a humanized monoclonal antibody (mAb) that selectively binds to calcitonin gene-related peptide (CGRP). Prior studies have proven that galcanezumab is efficacious, safe, and well-tolerated for the preventive treatment of episodic migraine (EM) and chronic migraine (CM) [11–13]. Post-hoc analyses from previous galcanezumab phase 3 clinical trials demonstrated a reduction in migraine headache days beginning the first week of treatment and showed a lower percentage of galcanezumab-treated patients reported a migraine headache day as early as the first day after treatment initiation [14, 15]. However, those prior phase 3 studies excluded patients with a history of failure to respond to an adequate trial of three or more classes of migraine preventive treatments as defined by the American Academy of Neurology/American Headache Society treatment guidelines Level (A) and (B) evidence [11–13].

Recently, in the CONQUER study, the efficacy and safety of galcanezumab was demonstrated in patients with episodic and chronic migraine who had previously failed to benefit from 2 to 4 standard-of-care migraine preventive medication categories [16]. At baseline, patients had 13.2 monthly migraine headache days. The galcanezumab group experienced 4.1 fewer monthly migraine headache days averaged across months 1–3 compared to 1.0 fewer monthly migraine headache days in the placebo group. This article aims to assess the onset of effect in the CONQUER population, a group of patients who theoretically may take longer to respond given their lack of benefit from multiple prior preventive treatments.

## Methods

### Study design

The current study includes post-hoc analyses from the CONQUER trial that assessed galcanezumab for the treatment of patients who had not benefited from 2 to 4 classes of migraine preventive treatments. Detailed description of the study design has been reported previously [16]. Briefly, CONQUER (NCT03559257; 10 September 2018 to 19 June 2019) was a phase 3b, multicenter, randomized, double-blind, placebo-controlled study. The study consisted of an initial screening period and washout of all migraine preventive treatments (3–30 days), a 1-month baseline period, a 3-month double-blind treatment period, and a 3-month open-label treatment period. Participants were randomized 1:1 to receive monthly subcutaneous placebo or galcanezumab 120 mg following a loading dose of 240 mg. Randomization was stratified by country and migraine frequency during the baseline period (low frequency EM, 4 to < 8 migraine headache days/month; high frequency EM, 8–14 migraine headache days/month and < 15 headache days/month; CM, ≥ 8 migraine headache days/month and ≥ 15 headache days/month). Participants were randomized by a computer-generated random sequence using an interactive web-response system. The study protocols were reviewed and approved by the institutional review board and conducted according to Good Clinical Practice and the Declaration of Helsinki guidelines. Patients provided written informed consent prior to initiating the study.

### Trial population

Eligible participants were 18 to 75 years of age with a diagnosis of migraine as defined by International Classification of Headache Disorders – Third edition [1], with a history of migraine for at least 1 year, and migraine onset prior to age 50. Eligible participants had to experience at least four migraine headache days and at least one headache-free day per month on average within the past 3 months. Patients were eligible if they had a history of documented failure of 2 to 4 standard-of-care migraine preventive medication categories in the past 10 years due to inadequate efficacy and/or safety/tolerability reasons. The medication categories were: propranolol or metoprolol, topiramate, valproate or divalproex, amitriptyline, flunarizine, candesartan, botulinum toxin A or B (if taken for CM), and medications approved by local regulatory agencies for prevention of migraine. Participants could continue the use of acute medications for migraine throughout the study. Patients with serious cardiovascular risk were not permitted to participate. An electronic diary was used to record migraine attacks. The full list of inclusion and exclusion criteria has been previously published [16].

### Outcomes measured

The primary endpoint of the study was the mean change from baseline in monthly migraine headache days averaged across months 1–3 of the double-blind treatment period. Key secondary endpoints included the percentage of patients who achieved a ≥50%, ≥75%, and 100% reduction in monthly migraine headache days averaged across months 1–3. The primary and all key secondary objectives were met.

Here, we report post-hoc analyses on early onset of effect assessed by month, week, and day using a sequential approach. Monthly onset of effect was defined as the earliest month at which significant improvement with galcanezumab compared to placebo was demonstrated and maintained at all subsequent months. If monthly onset was observed in a particular month, weekly onset of effect was assessed within that month. Weekly onset was defined as the initial week at which galcanezumab was superior to placebo and maintained superiority at all subsequent weeks during that month. If weekly onset was achieved, onset of effect at daily intervals within that week was assessed. Daily onset was defined as the initial day post-injection when a significantly smaller proportion of patients on galcanezumab experienced a migraine headache compared to placebo and maintained superiority for each subsequent day during that week.

The proportion of patients achieving ≥50%, ≥75%, and 100% response rates during months 1, 2, 3 and weeks 1, 2, 3, 4 of month 1 were also assessed. Similar to the sequential approach used for the migraine headache day reduction, response rates were first assessed monthly with the onset of response defined as the first month at which galcanezumab separated from placebo and maintained that response at all subsequent months. If onset of response occurred at a particular month, it was then assessed at weekly intervals within that month. Weekly onset of response was defined as the first week at which galcanezumab separated from placebo and maintained that response at all subsequent weeks during that month.

### Statistical analyses

These analyses were performed in the total intent-to-treat population, which included all patients who were randomized and received at least one dose of study drug. Change from baseline in number of monthly and weekly migraine headache days was assessed by mixed model with repeated measures using all the longitudinal observations at each post-baseline visit. The model included treatment, pooled country (North America, Europe, and Asia), month (or week for weekly migraine headache days), and treatment-by-month (or week for weekly migraine headache days) interaction as well as the continuous covariates baseline value and baseline value-by-month (or week for weekly migraine headache days). Least-square (LS) mean reduction in monthly (or weekly) migraine headache days with standard error (SE) as well as treatment effect difference among the 4 weeks within each month were estimated.

In this analysis, the data from the baseline month were normalized to a 1-week baseline for comparison with those of weeks 1 through 4 of the post-baseline period. Each week of the dosing interval started with the week immediately after dosing (“week 1”) and ended with the week immediately prior to the next dose (“week 4”).

Generalized linear mixed model was used to estimate the proportion of patients with migraine headache days in the first week of treatment, and patients achieving ≥50%, ≥75%, and 100% response rates by month and week. The model for monthly interval response outcomes included fixed, categorical effect of treatment category, month, treatment-by-month interaction and a continuous effect of baseline monthly migraine headache days. A similar covariate list was implemented for weekly interval response outcomes. Unstructured covariance matrix was implemented to measure the correlation among the repeated measures obtained on the same individuals in mixed models.

The approach to missing electronic diary data assumed that the rate of migraine headaches per day was the same for days with missing and non-missing electronic diary days. If the post-baseline diary compliance rate for a monthly interval was ≤50%, then all endpoints to be derived from the electronic diary data for that 1-month period were considered missing.

All statistical tests conducted were two-sided and *p*-values ≤ 0.05 were assumed to be statistically significant. No adjustments were made for multiple comparisons. Analyses were implemented using SAS Enterprise Guide 7.2 (SAS Institute, Cary, NC).

## Results

### Patient demographics and baseline disease characteristics

A total of 462 patients were randomized to be treated with placebo (*N* = 230) or galcanezumab 120 mg (*N* = 232). The study population had an average age of 46 years, was mostly female (86%), and Caucasian (82%). Of the participants, 58% of patients had EM and 42% of patients had CM. The average duration of migraine disease was 23 years. The average age of these patients was slightly older and their duration of migraine diagnosis was longer compared to those who enrolled in the galcanezumab pivotal phase 3 episodic and chronic migraine trials (EVOLVE-1, EVOLVE-2, and REGAIN) [11–13]. The average number of monthly migraine headache days at baseline was 13.2 and the average number of weekly migraine headache days was 3.1. On average, participants had not benefited from three individual preventive medications in the past 10 years. A total of 226 patients treated with placebo and 225 patients treated with galcanezumab 120 mg completed the study. All baseline demographics and disease characteristics were similar between treatment groups (Table 1).

**Table 1:** Patient demographics and baseline disease characteristics

Characteristic	PBO (***N*** = 230)	GMB 120 mg (***N*** = 232)
Age, years, mean (SD)	45.7 (12.3)	45.9 (11.3)
Female, n (%)	202 (87.8)	195 (84.1)
Race (*N* = 223), n (%)		
White	182 (81.6)	183 (81.7)
Asian	35 (15.7)	37 (16.5)
Black or African American	2 (0.9)	3 (1.3)
Duration of migraine illness, years, mean (SD)	23.8 (13.9)	22.7 (13.2)
Monthly migraine headache days, mean (SD)	13.0 (5.7)	13.4 (6.1)
Weekly migraine headache days,^a^ mean (SD)	3.0 (1.3)	3.1 (1.4)
Total number of failed individual preventive medications in past 10 years,^b^ mean (SD)	3.3 (1.7)	3.3 (1.6)

*PBO* Placebo. *GMB* Galcanezumab. *SD* Standard deviation. *N* Number of intent-to-treat patients. *n* Number of patients within each specific category

^a^Based on baseline monthly migraine headache days and normalized to a weekly range

^b^Based on any medications taken for migraine prevention in the past 10 years; not limited to the qualifying standard-of care treatments specified in the inclusion criteria. Medication failure was defined as discontinuation due to no response, inadequate response, or safety/tolerability event. Contraindications did not count as medication failures

### Reduction in migraine headache days by month, week, and day

**Fig. 1:**
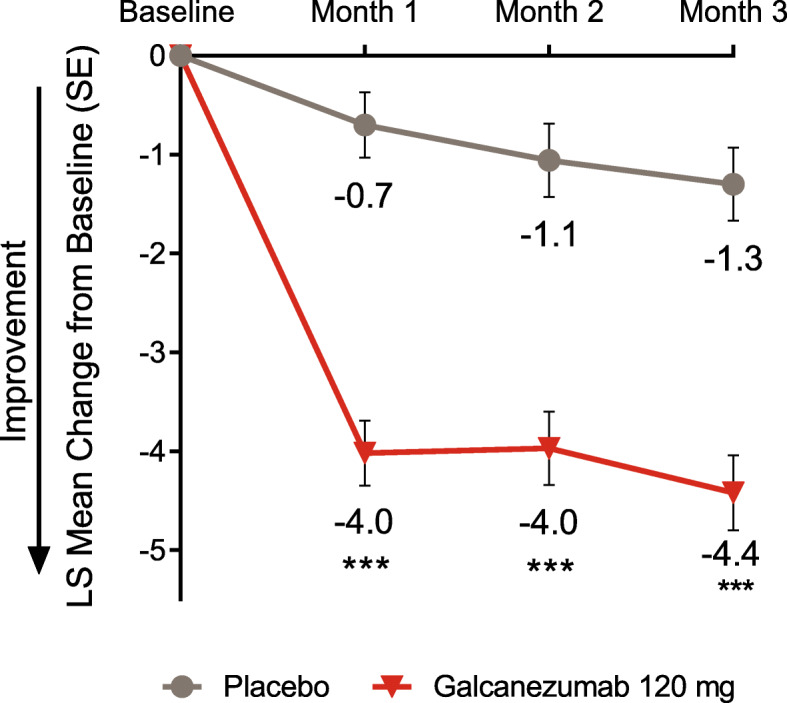
LS mean change from baseline in number of monthly migraine headache days. *** *p* ≤ 0.0001 vs placebo. *LS* least-squares. *SE* standard error

Galcanezumab-treated patients had a significant reduction in mean monthly migraine headache days starting at month 1 and continuing through month 3 compared to placebo (all *p* ≤ 0.0001, Fig. 1). At month 1, the LS mean change from baseline (SE) in number of migraine headache days was − 4.0 (0.3) for the galcanezumab group vs − 0.7 (0.3) for the placebo group.

**Fig. 2:**
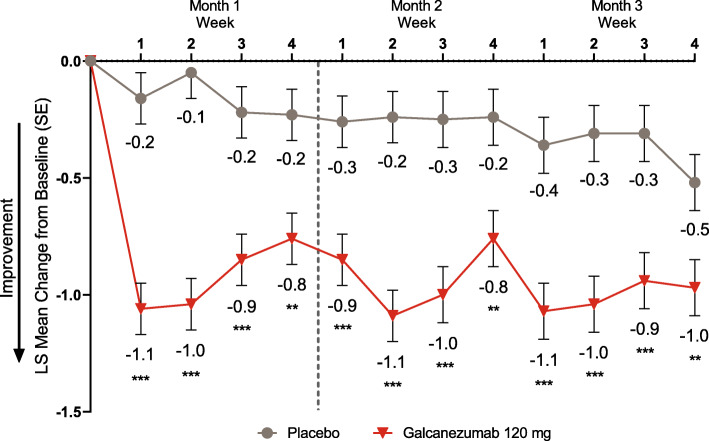
LS mean change from baseline in number of weekly migraine headache days. ** *p* < 0.01, *** *p* ≤ 0.0001 vs placebo. *LS* least-squares. *SE* standard error. Dotted line separates month 1, which was used in the onset analysis, from remaining months

Because month 1 was identified as the earliest month of onset of effect, weekly analyses were conducted for each week within month 1. Weekly migraine headache days were significantly reduced in galcanezumab-treated patients starting at week 1 and continuing for all subsequent weeks of month 1 compared to placebo (all *p* < 0.01, Fig. 2). At week 1, the LS mean change from baseline (SE) in number of migraine headache days was − 1.1 (0.1) for the galcanezumab group vs − 0.2 (0.1) for the placebo group. The mean difference in weekly migraine headache days between galcanezumab and placebo decreased from week 1 to week 4, week 2 to week 4, and week 2 to week 3 within month 1 (*p* < 0.05, Fig. 2), but there remained a statistically significant difference from baseline between the two groups. To determine if the trend persisted in months 2 and 3, the difference in weekly migraine headache days between galcanezumab and placebo was calculated among all 4 weeks in the latter 2 months and no evidence of significant reduction in efficacy towards the end of the month was observed (all *p* > 0.05, Fig. 2).

**Fig. 3:**
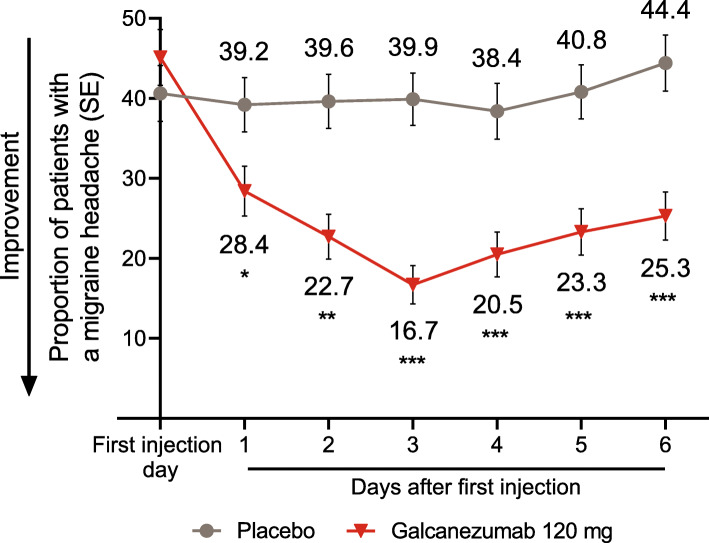
Estimated proportion of patients with a migraine headache on each day of the first week after the first injection in month 1. * *p* < 0.05, ** *p* < 0.01, *** *p* ≤ 0.0001 vs placebo. *SE* standard error

Because week 1 was identified as the earliest week of onset of effect, further analyses for each day of the first week of treatment were conducted. A smaller proportion of galcanezumab-treated patients compared to placebo had a migraine headache beginning the first day after the first injection (28.4% vs 39.2%) and each subsequent day during week 1 (all *p* < 0.05, Fig. 3). Thus, onset of effect for galcanezumab was determined to occur the first day following the initial injection.

### Monthly and weekly response rates

**Fig. 4:**
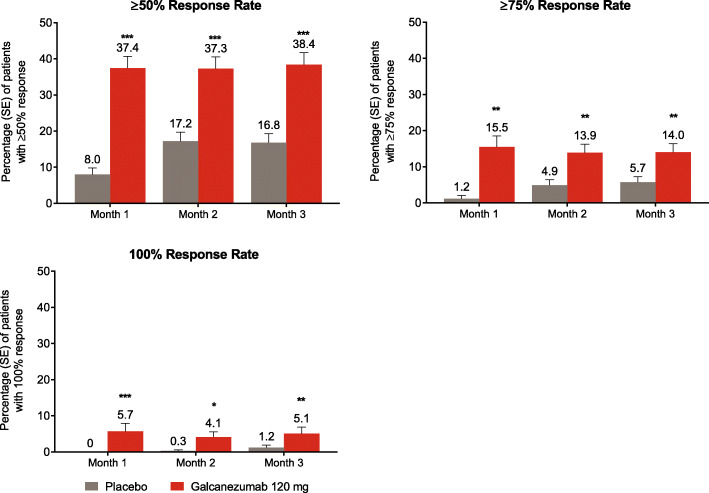
Estimated mean percentage of patients with ≥50%, ≥75%, and 100% reduction from baseline in monthly migraine headache days at months 1–3. * *p* < 0.05, ** *p* < 0.01, *** *p* ≤ 0.0001 vs placebo. *SE* standard error

A greater percentage of galcanezumab-treated patients reached a significant ≥50%, ≥75%, and 100% reduction from baseline in monthly migraine headache days compared to placebo starting at month 1 and continuing through month 3 (all *p* < 0.05, Fig. 4). Over twice as many patients treated with galcanezumab achieved ≥50% and ≥75% response compared to placebo-treated patients at each month, and over four times as many patients treated with galcanezumab achieved 100% response compared to placebo at each month.

**Fig. 5:**
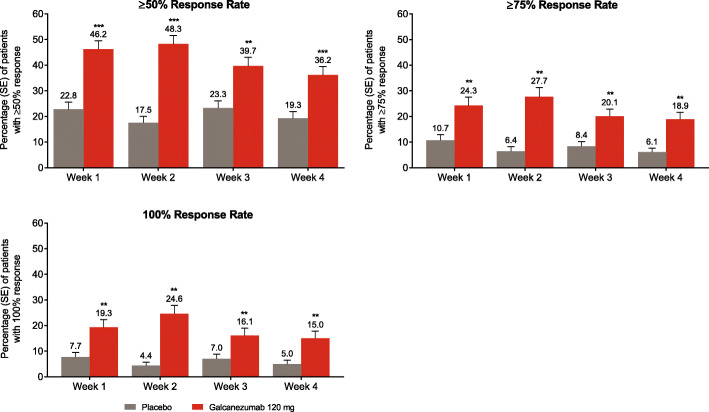
Estimated mean percentage of patients with ≥50%, ≥75%, and 100% reduction from baseline in weekly migraine headache days at weeks 1–4 of month 1. ** *p* < 0.01, *** *p* ≤ 0.0001 vs placebo. *SE* standard error

Because month 1 was identified as the earliest month of achieving these response rates, weekly analyses were conducted. A greater percentage of galcanezumab-treated patients reached a significant ≥50%, ≥75%, and 100% reduction from baseline in weekly migraine headache days compared to placebo starting at week 1 and continuing through week 4 of month 1 (all *p* < 0.01, Fig. 5). Over 1.5 times as many patients treated with galcanezumab achieved ≥50% response compared to placebo at each week, and over twice as many patients treated with galcanezumab achieved ≥75% and 100% response compared to placebo at each week.

## Discussion

Patients rate efficacy, including early onset of effect, as highly important when choosing a migraine preventive medication [10]. Patient preference is an important consideration when selecting a therapy because it can lead to improved adherence, enhanced patient satisfaction, and reduced disability [4, 10]. Prior analyses have demonstrated early onset of effect with galcanezumab in patients with EM and CM, but it was previously unknown whether this same effect held true in patients who had not previously benefited from multiple preventive medications, a group of patients who theoretically may take longer to respond.

This post-hoc analysis from CONQUER revealed that individuals for whom at least two prior migraine preventive medications had not provided benefit experienced early onset of effect. Galcanezumab treatment reduced mean monthly migraine headache days beginning at month 1 and this benefit persisted during months 2 and 3. Reduction in weekly migraine headache days in galcanezumab-treated patients began at week 1 and continued during weeks 2 through 4 of month 1. Additionally, a significantly lower percentage of patients had a migraine headache on the first day after galcanezumab treatment and at each subsequent day during week 1 compared to placebo. While there was some variability in the magnitude of therapeutic gain from galcanezumab among the 4 weeks of month 1, this was not seen in later months and there was no evidence of significant reduction in efficacy towards the end of months 2 and 3. Moreover, reduction in weekly migraine headache days from baseline remained superior for galcanezumab vs placebo in each week of every month.

Early onset of effect was also demonstrated via response rates. A significantly greater percentage of galcanezumab-treated patients experienced a ≥50%, ≥75%, and 100% reduction in monthly migraine headache days compared to placebo beginning at month 1 and continuing in months 2 and 3. A significantly greater percentage of patients in the galcanezumab treatment arm also experienced a ≥50%, ≥75%, and 100% reduction in weekly migraine headache days compared to placebo beginning at week 1 and continuing for all subsequent weeks of month 1. These early response rates support the onset analysis based on reduction in migraine headache days and provides clinical meaningfulness [17–20].

These findings may be related to the pharmacokinetic profile of galcanezumab. Galcanezumab binds to CGRP and inhibits its ability to bind to the CGRP receptor [21]. The average time to peak serum galcanezumab concentration is 5 days after the initial dose, and galcanezumab reaches therapeutic steady state following the loading dose [22]. This is advantageous over existing oral migraine preventive medications that require daily dosing and long titration schedules before reaching a therapeutic dose [6].

Early onset of effect along with favorable tolerability and lack of titration may be advantageous for patient adherence and improvement of outcomes over time [9, 10]. However, not all patients will experience early onset with galcanezumab, and this early response is not necessarily indicative of a response later in the treatment course. Prior analyses have shown that patients with EM or CM treated with galcanezumab who do not respond in the first 2 months of treatment have a reasonable likelihood of improvement in successive months [23]. The American Headache Society recommends trialing monthly administered CGRP mAbs for 3 months before deciding whether they are efficacious for an individual patient and if therapy should be continued [4].

There are some limitations that should be considered when interpreting these results. Patients were mostly female, white, and middle-aged, which is reflective of the migraine population but may not be generalizable to everyone. This study excluded patients who did not benefit from more than 4 migraine preventive medication categories and those who did not experience any headache-free days. Further, migraine frequency is variable and weekly assessment of migraine frequency may not be as stable as monthly assessments, especially for patients with lower frequency EM. The study was not powered for the complete set of analyses presented in this manuscript. However, the response rates and consistency of early onset findings across this and prior galcanezumab post-hoc analyses lends credibility to the conclusions.

## Conclusion

Galcanezumab 120 mg monthly (with a 240 mg loading dose) achieved early onset of effect in migraine headache day reduction beginning the first day after initial injection. Galcanezumab was also superior to placebo in ≥50%, ≥75%, and 100% response rates starting at week 1. These data further support the efficacy of galcanezumab in patients who have not benefited from multiple prior preventive treatments.

## Data Availability

Lilly provides access to all individual participant data collected during the trial, after anonymization, with the exception of pharmacokinetic or genetic data. Data are available to request 6 months after the indication studied has been approved in the United States and European Union and after primary publication acceptance, whichever is later. No expiration date of data requests is currently set once data are made available. Access is provided after a proposal has been approved by an independent review committee identified for this purpose and after receipt of a signed data sharing agreement. Data and documents, including the study protocol, statistical analysis plan, clinical study report, blank or annotated case report forms, will be provided in a secure data sharing environment. For details on submitting a request, see the instructions provided at www.vivli.org.

## Abbreviations

CGRP: Calcitonin gene-related peptide
CM: Chronic migraine
EM: Episodic migraine
GMB: Galcanezumab
LS: Least-squares
mAb: Monoclonal antibody
n: Number of patients within each specific category
N: Number of intent-to-treat patients
PBO: Placebo
SD: Standard deviation
SE: Standard error
